# Successful Treatment of Refractory Status Asthmaticus With Veno-Venous Extracorporeal Membrane Oxygenation (VV ECMO): A Case Report

**DOI:** 10.7759/cureus.78672

**Published:** 2025-02-07

**Authors:** Llewellyn Tan, Imani Thornton

**Affiliations:** 1 Anesthesiology, HCA Florida Westside Hospital, Plantation, USA

**Keywords:** asthma, bronchospasm, ecmo, refractory status asthmaticus, respiratory acidosis, veno-venous ecmo

## Abstract

Refractory status asthmaticus is an extreme form of asthma exacerbation characterized by hypoxemia, hypercarbia, and secondary respiratory failure and is considered a medical emergency. Mechanical ventilation and maximal medical therapy are often required for treatment, but they frequently fail to provide a resolution. Extracorporeal membrane oxygenation (ECMO), however, has increasingly been used as an unconventional treatment modality if all others fail. Here, we report the case of a 46-year-old male who presented to the emergency department (ED) with shortness of breath. The patient demonstrated signs and symptoms of refractory status asthmaticus with significant wheezing and accessory muscle use on physical exam and failed to respond to maximal therapy, including bronchodilators, high-dose steroids, magnesium sulfate, heliox, noninvasive ventilation, inhaled anesthetics, and mechanical ventilation. Ultimately the patient was placed on veno-venous (VV) ECMO, remained in the intensive care unit (ICU) for seven days, and was discharged to home on day 11 post-ICU admission.

## Introduction

Refractory status asthmaticus is a rare but severe and fatal form of bronchospasm that fails to respond to conventional asthma therapies. The prevalence is about 3.6% of asthmatic adults [1]. Approximately 2%-4% of patients hospitalized for status asthmaticus require mechanical ventilation, and of those patients, 7% do not survive [2,3]. Extracorporeal membrane oxygenation (ECMO) has been recognized as a viable option for patients with refractory respiratory failure, but no clear guidelines exist to delineate its use in patients with refractory status asthmaticus. A recent retrospective cohort study of patients admitted with severe asthma exacerbation requiring mechanical ventilation found that 127 of 13,587 patients (0.9%) were supported with ECMO. Of those patients, the survival rate was reported to be greater than 80% [4].

We report a case of successful use of veno-venous (VV) ECMO in a patient with refractory status asthmaticus who failed to respond to all other treatments in the intensive care unit (ICU). Risk factors that have been associated with increased risk of refractory status asthmaticus include history of poor asthma control, late presentation despite symptom onset, smoking, psychiatric illness, and preexisting cardiopulmonary conditions [5]. The pathophysiology of status asthmaticus involves inflammation of the lower airways leading to airway obstruction, ventilation-perfusion mismatch, hypoxemia, hypercarbia, and respiratory muscle fatigue. Inflammation is marked by mast cell degranulation after allergen exposure and the release of inflammatory mediators such as histamine, interleukins, and prostaglandins. Bronchospasm occurs due to smooth muscle activation which causes airflow obstruction and is worsened by hypersecretion of mucus. Respiratory rate increases to compensate for impaired ventilation, but prolonged expiratory times lead to lung hyperinflation and worsening of symptoms [5]. The management of status asthmaticus may include β2-agonists, anticholinergics, corticosteroids, magnesium sulfate, heliox, noninvasive ventilation, mechanical ventilation with sedation and paralytics, and ECMO. The approach to management often depends on the timing of symptomatology, the ability to reverse inflammation and bronchoconstriction with initial treatment, the degree of respiratory failure, laboratory studies, and trigger investigation [6].

## Case presentation

We report the case of a 46-year-old male who presented to the ED with a chief complaint of shortness of breath and wheezing. The patient had a past medical history of asthma and polysubstance abuse. The patient had no known allergies, no surgical history, and took no home medications. Social history was significant for active smoking of 1 pack per day. At the time of presentation, patient vitals included a heart rate (HR) of 94 beats per minute (bpm), respiratory rate (RR) of 22 breaths per minute, blood pressure (BP) of 186/120 mmHg with a mean arterial pressure (MAP) of 142, and oxygen saturation (SpO2) of 95% on room air. Upon physical exam, it was noted that the patient had physiology concerning status asthmaticus, including decreased bilateral breath sounds, diffuse wheezing, accessory muscle use, labored breathing, and inability to speak in full sentences. The patient was alert and oriented to person, place, and time, and the rest of the physical examination was within normal limits.

**Figure 1:**
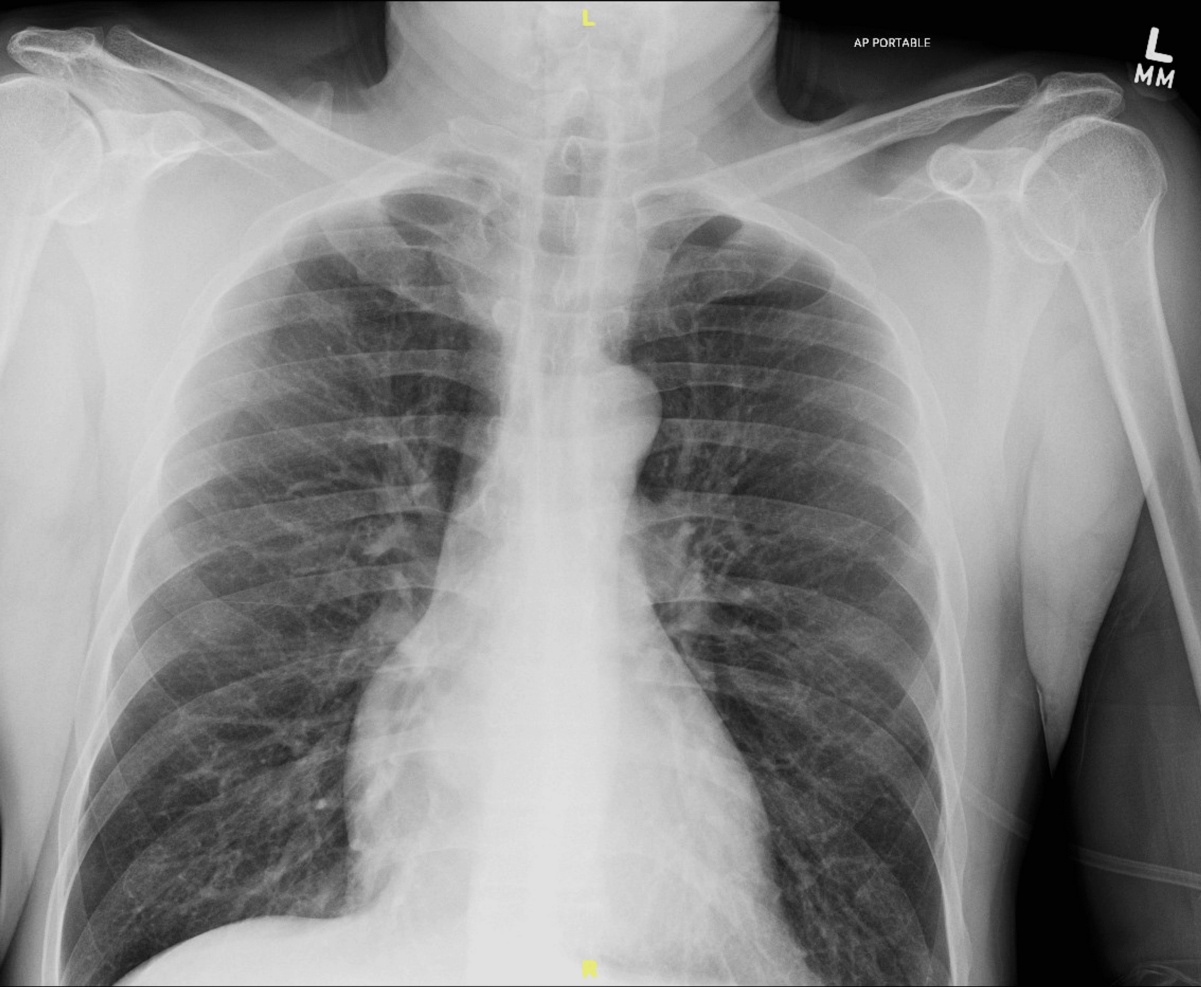
A portable supine chest anteroposterior X-ray showing pulmonary hyperinflation with air trapping.

Initial chest X-ray demonstrated pulmonary hyperinflation with air trapping, which is demonstrated in Figure 1. A urine toxicology screen was positive for cocaine, methamphetamine, and marijuana. The patient was initially placed on continuous positive airway pressure (CPAP) due to respiratory distress and received three doses of ipratropium bromide 0.5 mg, two doses of albuterol 1.25 mg, intravenous methylprednisolone 125 mg, and intravenous magnesium sulfate 2 g. Despite these interventions, the patient failed to improve and required intubation when he could no longer tolerate CPAP. The arterial blood gas (ABG) immediately after intubation revealed a pH of 6.86, PCO2 171 mmHg, PO2 368 mmHg, and HCO3 30 mmol/L, demonstrating hypercapnia with severe respiratory acidosis. Serial ABGs and ventilator parameters throughout the patient’s hospital stay are shown in Table 1. The patient was admitted to the ICU and started on multiple infusions including propofol and versed for sedation, bicarbonate for acidosis, cisatracurium for paralysis, and epinephrine and ketamine for systemic bronchodilation. The patient was also receiving DuoNeb (Viatris Inc., Canonsburg, PA) treatments every two hours, Solu-Medrol (Pfizer Inc., New York, NY) 80 mg every six hours, continuous albuterol, and heliox via the ventilator. Ventilator settings consisted of assist control mode, tidal volume of 500 mL, RR of 20, inspiratory-to-expiratory (I:E) ratio of 1:2, FiO2 of 100%, and positive end-expiratory pressure (PEEP) of 5 cmH2O. Auto-PEEP was noted on the ventilator; therefore, RR was decreased to 14, the I:E ratio was adjusted to 1:9, and PEEP was increased to 10 cmH2O. Despite all measures, end-tidal CO2 (ETCO2) on the ventilator remained in the 65-70 mmHg range, and peak airway pressure stayed elevated at 39 mmHg. Repeat ABG revealed a pH of 7.19, pCO2 91.2 mmHg, pO2 180.5 mmHg, and HCO3 35.0 mmol/L, showing only mild improvement.

**Table 1:** Ventilator parameters and serial arterial blood gases at admission and throughout the treatment course. TV, tidal volume; RR, respiratory rate; PIP, peak inspiratory pressure; PEEP, positive end-expiratory pressure; FiO2, fraction of inspired oxygen; PaCO2, partial pressure of carbon dioxide; PaO2, partial pressure of oxygen; SaO2, oxygen saturation

	Immediately after intubation	Six hours after intubation	Immediately before ECMO	Immediately after ECMO	Six hours after ECMO	Before ECMO decannulation
Ventilator parameters						
TV (mL)	500	500	475	500	450	400
RR (breaths/minute)	20	14	14	10	10	14
PIP (cmH2O)	39	39	51	54	30	15
PEEP (cmH2O)	5	10	10	10	10	5
FiO2 (%)	100	100	100	100	80	40
Arterial blood gas						
pH	6.86	7.19	7.20	7.35	7.35	7.37
PaCO2 (mmHg)	171	91.2	91.7	57.7	35	50
PaO2 (mmHg)	368	180	87	431	290	476
HCO3 (mmol/L)	30	35	35.8	32	19	28.6
SaO2 (%)	100	99.1	93.3	100	99.3	99.7

Cardiothoracic surgery was consulted and scheduled the patient for VV ECMO cannula placement the same day due to persistent severe hypercapnia. The left and right femoral veins were cannulated as an inflow and outflow to the ECMO circuit, respectively. Transesophageal echocardiography (TEE) was employed to confirm successful cannula placement. There was no valvular pathology, pericardial effusion, or left ventricular dysfunction noted on the TEE exam. After initiation of ECMO, repeat serial ABGs showed marked improvement, as shown in Table 1. Ventilator settings were reduced to a tidal volume of 450 mL, RR of 10, and FiO2 of 80%. Peak pressures decreased to 30 mmHg, and the frequency of DuoNeb administration was reduced from every two hours to every three hours. Systemic anticoagulation was maintained with bivalirudin at 2 mcg/kg/minute to maintain a partial thromboplastin time between 40 and 60 seconds.

VV ECMO was continued for six days, and the previously mentioned infusions were continued throughout the ECMO period. The ventilator settings continued to be weaned until they reached the minimum. On the sixth day, the patient was decannulated from ECMO and extubated nine hours later. The patient remained stable in the ICU for two days post-decannulation and was subsequently discharged home on postoperative day 11.

## Discussion

Refractory status asthmaticus is defined as a severe acute asthma exacerbation that is unamenable to maximum pharmacological therapy and mechanical ventilation. It is very rare with a prevalence of about 3.6% among asthmatic adults in the United States [1]. If left untreated, refractory status asthmaticus often results in respiratory failure, profound hypercapnia, hypoxemia, pneumothorax, pneumonia, cardiac arrest, and death [5,7,8]. The diagnosis of refractory status asthmatics is clinical and made when a patient presents with severe respiratory distress, persistent wheezing, and significant dyspnea despite receiving standard asthma medications and ventilatory therapy. Early use of VV ECMO has emerged as a successful form of treatment in these cases [9].

Refractory status asthmaticus remains an uncommon indication for VV ECMO. Although VV ECMO has primarily been used to treat respiratory failure due to acute lung injury (ALI) and acute respiratory distress syndrome (ARDS), no clear evidence-based guidelines exist for its use in refractory status asthmaticus [8]. The current published literature on the use of VV ECMO in adult refractory status asthmaticus is limited to one retrospective cohort study [9] and a few case reports [10-14]. However, it is interesting to note that most of these studies show positive outcomes with VV ECMO use in treating refractory status asthmaticus when mechanical ventilation fails. For example, the retrospective cohort study found that among 24 patients who had status asthmaticus as a primary indication for extracorporeal life support (ECLS), 20 of them (83.3%) survived hospital discharge, compared to 50.8% of non-asthmatics. Thus, it concluded that status asthmaticus, as an indication for ECLS in adult respiratory failure, appeared to be associated with greater survival than other indications for ECLS. This may be due to the reversibility of status asthmaticus compared with other respiratory conditions. In addition, being placed on ECMO minimizes barotrauma and dynamic hyperinflation that are associated with prolonged mechanical ventilation.

This case serves as another example of the benefits of using VV ECMO to treat status asthmaticus when other conventional therapies fail. It provided a bypass that allowed oxygenation of blood and removal of CO2, providing pulmonary and hemodynamic support until the bronchospasm subsided. Early recognition in identifying refractory status asthmaticus is also important based on patient risk factors. In this case, the patient had a history of polysubstance use and was not taking any asthma medications, both of which are known factors to increase the risk of refractory status asthmaticus. Knowledge of these risk factors could enable providers to anticipate the possibility of refractory status asthmaticus in patients and consider the use of VV ECMO earlier. The use of VV ECMO, however, is not without complications. A review consisting of six retrospective studies revealed a mortality rate of 17% (13%-20%), pooled risk of bleeding of 22% (7%-37%), mechanical complications in 26% (21%-31%), infection in 8% (0-21%), and pneumothorax in 4% (2%-6%) of patients with status asthmaticus requiring ECMO as rescue therapy after failing ventilatory support [15]. Thus, providers must be aware and adept at managing complications while weighing the risks versus benefits. As for our case, the patient had persistent severe respiratory acidosis, hypercapnia, and bronchospasms despite multiple pharmacological therapies and mechanical ventilation, which necessitated VV ECMO as an unconventional but last line of treatment. The patient eventually recovered, and no complications associated with VV ECMO were observed.

This case highlights the importance of VV ECMO in treating refractory status asthmaticus. Rapid recognition and early treatment of this complication can improve outcomes and prevent morbidity and mortality in these patients. Further research is needed to delineate clear evidence-based guidelines on when to initiate VV ECMO on these patients. Unfortunately, VV ECMO is not widely available in most institutions due to the significant equipment costs and the need for trained personnel. High-quality trials supporting the use of VV ECMO for status asthmaticus are important to further increase its accessibility to patients in the future.

## Conclusions

This case report reinforces the importance of VV ECMO in successfully treating refractory status asthmaticus. VV ECMO has been shown to improve survival in select cases when patients fail to respond to medical treatments and ventilatory support. In our case, the patient was managed with early placement of VV ECMO and discharged home after seven days in the ICU.
